# Exploring Wearables to Focus on the “Sweet Spot” of Physical Activity and Sleep After Hospitalization: Secondary Analysis

**DOI:** 10.2196/30089

**Published:** 2022-04-27

**Authors:** S Ryan Greysen, Kimberly J Waddell, Mitesh S Patel

**Affiliations:** 1 Section of Hospital Medicine University of Pennsylvania Philadelphia, PA United States; 2 Philadelphia Corporal Michael Crescenz Veterans Medical Center Philadelphia, PA United States; 3 Ascension Health St Louis, MO United States

**Keywords:** sleep, physical activity, hospitalization, wearables, health care, digital health, patient reported outcomes, hospital

## Abstract

**Background:**

Inadequate sleep and physical activity are common during and after hospitalization, but their impact on patient-reported functional outcomes after discharge is poorly understood. Wearable devices that measure sleep and activity can provide patient-generated data to explore ideal levels of sleep and activity to promote recovery after hospital discharge.

**Objective:**

This study aimed to examine the relationship between daily sleep and physical activity with 6 patient-reported functional outcomes (symptom burden, sleep quality, physical health, life space mobility, activities of daily living, and instrumental activities of daily living) at 13 weeks after hospital discharge.

**Methods:**

This secondary analysis sought to examine the relationship between daily sleep, physical activity, and patient-reported outcomes at 13 weeks after hospital discharge. We utilized wearable sleep and activity trackers (Withings Activité wristwatch) to collect data on sleep and activity. We performed descriptive analysis of device-recorded sleep (minutes/night) with patient-reported sleep and device-recorded activity (steps/day) for the entire sample with full data to explore trends. Based on these trends, we performed additional analyses for a subgroup of patients who slept 7-9 hours/night on average. Differences in patient-reported functional outcomes at 13 weeks following hospital discharge were examined using a multivariate linear regression model for this subgroup.

**Results:**

For the full sample of 120 participants, we observed a “T-shaped” distribution between device-reported physical activity (steps/day) and sleep (patient-reported quality or device-recorded minutes/night) with lowest physical activity among those who slept <7 or >9 hours/night. We also performed a subgroup analysis (n=60) of participants that averaged the recommended 7-9 hours of sleep/night over the 13-week study period. Our key finding was that participants who had both adequate sleep (7-9 hours/night) and activity (>5000 steps/day) had better functional outcomes at 13 weeks after hospital discharge. Participants with adequate sleep but less activity (<5000 steps/day) had significantly worse symptom burden (z-score 0.93, 95% CI 0.3 to 1.5; *P*=.02), community mobility (z-score –0.77, 95% CI –1.3 to –0.15; *P*=.02), and perceived physical health (z-score –0.73, 95% CI –1.3 to –0.13; *P*=.003), compared with those who were more physically active (≥5000 steps/day).

**Conclusions:**

Participants within the “sweet spot” that balances recommended sleep (7-9 hours/night) and physical activity (>5000 steps/day) reported better functional outcomes after 13 weeks compared with participants outside the “sweet spot.” Wearable sleep and activity trackers may provide opportunities to hone postdischarge monitoring and target a “sweet spot” of recommended levels for both sleep and activity needed for optimal recovery.

**Trial Registration:**

ClinicalTrials.gov NCT03321279; https://clinicaltrials.gov/ct2/show/NCT03321279

## Introduction

Inadequate sleep and physical activity are common during and after hospitalization, but little is known about how long these disruptions continue after discharge and the consequences on functional outcomes such as mobility and overall physical health. Disruptions of normal patient activities (such as sleep, mobility, nutrition, self-care) may be traumatic for acutely ill patients and lead to a high-risk, generalized condition that has been described as “posthospital syndrome” [1-3]. The relationship between sleep and physical activity is particularly complex among patients who have been recently hospitalized [4-7]. Improving our understanding of how posthospitalization sleep and physical activity impact longer-term patient-reported outcomes will directly benefit future interventions designed to improve functional outcomes and return to normal life activities. One major challenge to studying posthospital syndrome and factors that impact sleep, physical activity, and patient-centered outcomes is the lack of data. Unlike at the hospital, the period of recovery at home after discharge is largely unobservable to providers [8-11]. Wearable sleep and activity trackers present an important opportunity to unobtrusively collect and study data during this critical and poorly understood period of care.

Previous work has examined the impact of disrupted in-hospital sleep and physical activity on short–term recovery [1-6], but much less is known about how posthospitalization sleep and physical activity impact patient-reported outcomes, especially beyond the 30-day window [12,13]. Similarly, although wearable devices have been deployed to study sleep at home, there have been very few applications of this technology to the postdischarge period specifically [14-16]. Furthermore, previous studies of the postdischarge period do not integrate patient-reported measures of sleep and activity with patient-measured wearable data to create a multidimensional, longitudinal view of this period. This lack of data on sleep and activity during the postdischarge period represents an important knowledge gap for optimizing the clinical care of hospitalized patients as well as an important opportunity for the field of mobile health (mHealth).

Given these gaps in the existing literature, we evaluated sleep and activity data from wearable devices worn by patients for 90 days (13 weeks) after hospital discharge. The purpose of this study was to examine the relationship between daily sleep and physical activity with 6 patient-reported functional outcomes at 13 weeks after hospital discharge. We used evidence-based recommendations to define ranges for healthy sleep as 7-9 hours per night [17,18] and for physical activity as over 5000 steps per day [19-21]. We explored the distribution of sleep and activity data from our study using these established parameters and then identified a subset of participants who met both guidelines for healthy sleep and activity. We defined this overlap of healthy sleep and activity as the “sweet spot” and hypothesized that this subset would have better functional outcomes compared with other participants.

## Methods

### Research Aims and Objectives

Our specific objectives for this study were to (1) observe the distribution of sleep and step data in our sample and describe any patterns, (2) explore differences between any observed patterns and functional outcomes (change in activities of daily living, symptom burden, physical health, quality of sleep, and life space), and (3) explore associations of functional outcomes with sleep and activity patterns using evidence-based guidelines for sleep and activity as an a priori subgroup. Our overarching hypothesis was that participants with both adequate sleep and activity would have better functional outcomes than those with only one (sleep vs activity).

### Overview of Parent Trial Enrollment, Study Design, and Outcome Measures

This was a secondary analysis from the Mobility and Outcomes for Validated Evidence Incentives Trial (MOVE IT; NCT #03321279). Full details of the primary trial have been previously reported [22,23]. Briefly, this study enrolled individuals ≥18 years of age who were admitted to a general medicine or oncology service at a single, urban, academic hospital. Participants were enrolled during their hospital admission, but the intervention began after discharge. Participants were first observed for 1 week after discharge to establish a baseline level of postdischarge activity in their home. Participants were then randomized to a 12-week intervention that examined the impact of gamification and social incentives on physical activity. Gamification is the use of game design elements, such as points and levels, to motivate behavior change (such as physical activity). Social incentives, such as peer support and collaboration, can augment gamification interventions by further motivating individuals to change behaviors based on social ties [24,25]. The goal of this study was to determine if an intervention with gamification and social incentives could increase physical activity (mean steps per day) after hospital discharge. Overall, the intervention arm did not have significantly higher physical activity than the control arm, which suggests that gamification and social incentives did not alter behavior patterns after discharge for this cohort as a whole.

In addition to steps per day (which was the primary outcome of the parent trial), we assessed patient-reported outcomes at baseline (enrollment) and at 13 weeks after hospital discharge. All participants completed standardized assessments that quantified basic and instrumental activities of daily living (scales by Katz et al [26] and Lawton and Brody [27]), mobility (Life Space Assessment [28]), symptom burden (Edmonton Symptom Assessment Survey [29]), physical health (Short-Form 12 physical component scale [PCS-12] [30]), and sleep quality (Pittsburgh Sleep Quality Index [PSQI] [31]).

### Ethical Considerations

The Institutional Review Board at the University of Pennsylvania approved this study (826974), and all participants provided informed consent. Additionally, to acknowledge the time and effort contributed by participants to this study, we offered US $300 for completing all the surveys required for the study. Furthermore, we monitored closely for adverse or unexpected events and provided clear contact information to participants to reach study staff and prompt responses (within 24 hours) to any questions or concerns raised. Four participants experienced a rash on their wrist where the device was worn and were provided with cloth-based replacement bands, which resolved the issue.

### Wearable Device and Research Platform Specifications

Daily steps and sleep were quantified via a wrist-worn wearable device (Withings Activité). The Activité is a wearable activity tracker designed to look and function like a wristwatch. The face of the device has hour, minute, and second hands like a traditional wristwatch with an additional dial in the center that indicates progress towards a preset step goal. The device is water-resistant to 50 meters/5 atmospheres and uses a traditional watch battery (CR2025), which does not require frequent charging and lasts about 8 months. These features help to increase consistent wear by users. Motion is detected by a high-precision MEMS 3-axis accelerometer and translated into steps or sleep using a proprietary algorithm. Synchronization between the device and user smartphone occurs automatically every 6 hours and is also triggered by each 1000 steps taken since the last sync. Previous studies by our group and others have demonstrated the reliability and validity of step counts from commercially available devices for activity studies [32], and the Activité has been validated against actigraphy and medical-grade pedometers [33].

We also leveraged the Way to Health platform, a National Institutes of Health (NIH)–funded research technology platform at the University of Pennsylvania that has been used for more than 150 clinical trials in 45 states using smartphones and wearable devices previous studies [25,34]. The Way to Health platform provides an easy way for data from participant wearable devices to be collected for research purposes through a secure server that tracks enrollment and automatically randomizes patients to intervention arms. The platform also has a messaging engine that can provide automated feedback to participants to increase the feasibility of studies (such as MOVE IT, the parent trial for this study) that use strategies such as gamification and social incentives.

### Step and Sleep Data

Step data calculated by summing the number of steps in each 24-hour period (12 am to 12 am). Step data were missing in 37.7% of the current sample. Step data were considered missing for any day the participant did not wear their device, sync their data to the Way to Health platform, or reach 1000 steps. We have used this method in prior work [24,25,35,36], because evidence indicates that daily step values ≤1000 may not represent full data capture (eg, wearing the device for only a few hours in that day) [37,38]. Sensitivity analyses were conducted in primary analyses of the parent trial to explore results without step values ≤1000; no significant differences were demonstrated when step values <1000 were included. We combined multiple imputations of step data using the mice package in R (version 3.4.0), which allows for participant random effects and combined results using the standard rules by Rubin [39]. This imputation approach has been used in our prior work [24,25,36,37].

Daily sleep was calculated by summing the number of sleep minutes between 12 pm and 12 pm the following day (24-hour period). Sleep data were missing in 39.8% of the current sample. Sleep data were considered missing if the participant did not wear or sync their device, did not wear the device at night, or registered an errant number. We used multiple imputation for missing sleep data with the classification and regression trees (CART) method, which we have used in previous studies [40].

### Statistical Analysis

To create our study sample for this paper from the original data set, we performed analyses in 2 steps. First, we tested for differences in the 6 patient-reported outcomes at 13 weeks by activity category (less active vs active) using all participants with available data at week 13 (n=120). Second, in an effort to disentangle the impact of sleep and physical activity on patient-reported outcomes, we then restricted the full sample to a subgroup of participants (n=60) who averaged the recommended 7-9 hours of sleep/night [17,18] over the intervention period.

The primary outcome for this analysis was the patient-reported outcomes on 6 standardized assessments administered at 13 weeks postdischarge. We calculated each participant’s average daily steps and total sleep minutes over the 12-week intervention period. Participants who averaged <5000 steps/day were classified as less active, and those who averaged >5000 steps/day were classified as active [19-21]. Because all standardized assessment measures used different scoring scales, we converted all scores to z-scores.

Since our outcomes were scores from clinical assessment scales (not binary “yes/no” outcomes), we used multivariate linear regression models with each outcome as a continuous primary (dependent) variable and activity category (less active or active) as the primary predictor variable. We also adjusted for other factors as covariates, which were selected because they were different between the 2 categories at baseline: age, gender, Charlson comorbidity index, and study arm. We tested for differences in the 6 patient-reported outcomes at 13 weeks between those who were less active and those who were active but averaged between 7 hours and 9 hours of sleep per night. All analyses were completed in R [41] with a *P*<.05 significance level.

## Results

**Figure 1:**
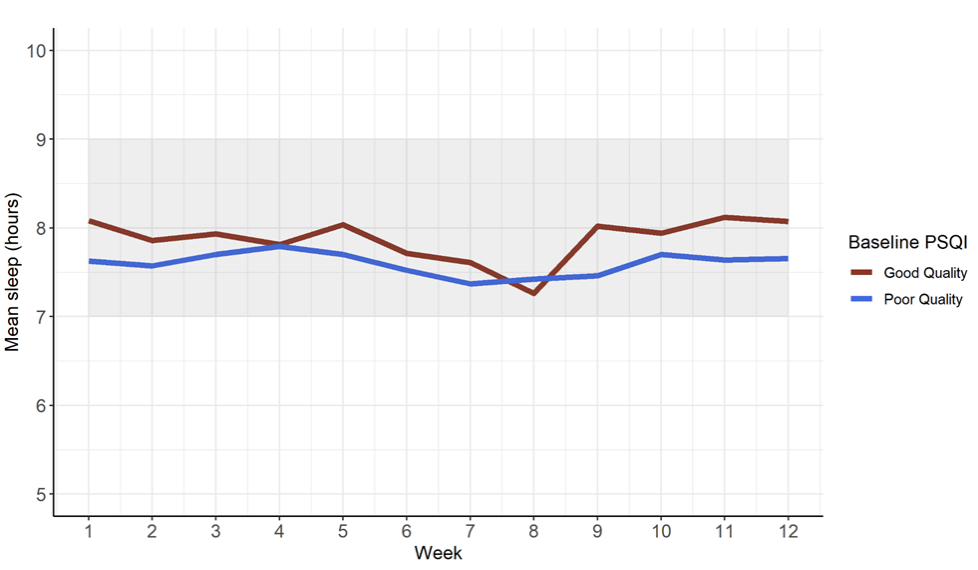
Mean daily sleep by baseline (prehospitalization) Pittsburgh Sleep Quality Index (PSQI) score.

**Figure 2:**
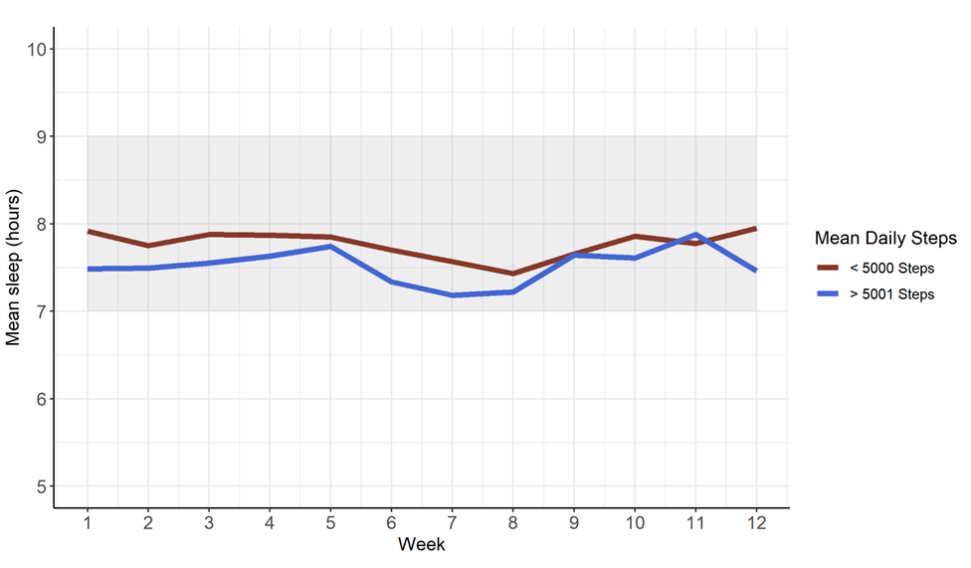
Mean daily sleep by level of physical activity after discharge.

Of the 232 participants in the primary trial, 120 had device-recorded sleep and step data over the entire study period and patient-reported follow-up survey data at week 13. As shown in Figure 1, patients who reported poor quality sleep (PSQI ≥5) in the month preceding hospitalization did not have different sleep patterns (mean 7.6, SD 1.8 hours per night) over the postdischarge period compared with those who had good quality sleep (mean 7.9, SD 1.7 hours per night). Similarly, we found no difference in sleep patterns for patients who were less physically active after discharge (≤5000 steps/day; mean 7.7, SD 2.0 hours per night) compared with those who were more active (>5000 steps/day; mean 7.5, SD 1.3 hours per night; Figure 2).

**Figure 3:**
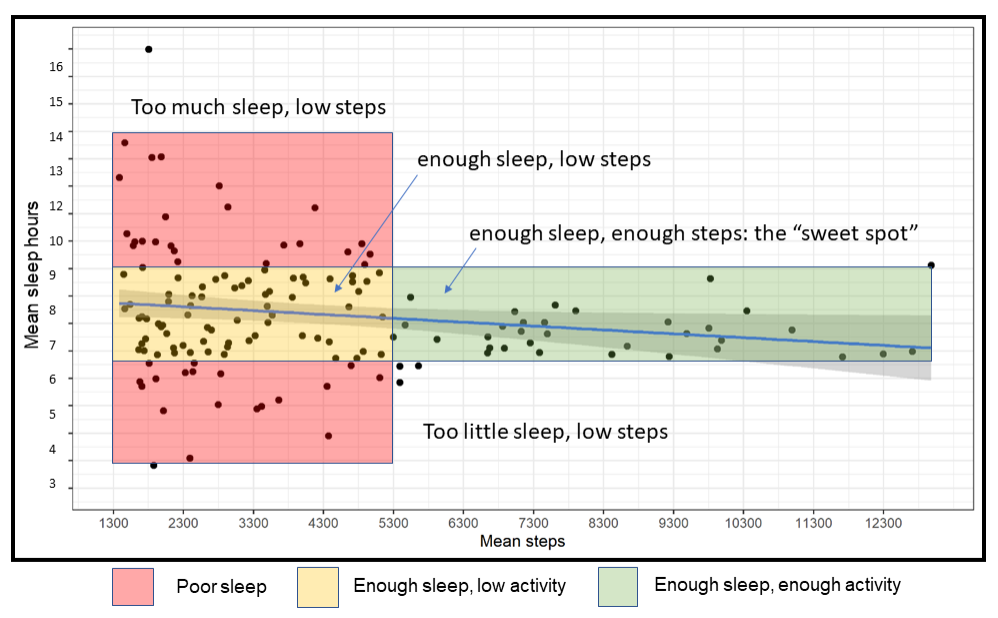
Mean daily sleep and mean daily activity distributions.

As shown in Figure 3, we plotted a single mean value for sleep (minutes) and mean value for activity (steps) for each patient over the entire postdischarge period. Although we observed a linear relationship between less sleep and more activity (–2 minutes of sleep/night per +1000 steps/day; blue line in Figure 3), on closer inspection using a lens of the “T-shaped” distribution between sleep and health, we identified 4 distinct groupings: low sleep (<7 hours) and low steps (<5000), high sleep (>9 hours) and low steps (<5000), recommended sleep (7-9 hours) and low steps, and recommended sleep with high steps (>5000) or “the sweet spot.”

To further explore these clusters, we first defined participants as “less active” (<5000 steps/day; n=83) versus “active” (≥5000 steps/day; n=37). Among the 120 participants, those who were less active reported significantly worse physical health (PCS-12 z-score –0.6, 95% CI –0.9 to –0.1; *P*=.004) compared with those who were active at 13 weeks after hospital discharge. This difference, however, was present at baseline with the less active group reporting worse physical health compared with the active group (z-score mean –0.18 less active vs –0.39 active, 95% CI 0.2 to 0.9, *P*=.003). There were no other differences in patient-reported outcomes among the full sample of 120 participants.

We then focused our analysis on a subgroup of 60 participants who averaged 7 hours to 9 hours of sleep per night over 13 weeks after discharge (see clusters represented by yellow and green boxes in Figure 3). Demographics from this subgroup analysis are reported in Table 1.

**Table 1:** Participant demographics and baseline assessments.

Characteristics and assessments			Less active (n=36)		Active (n=24)		*P* value
Age (years), mean (SD)			41.7 (13.5)		29.8 (10.9)		<.001
Female gender, n (%)			29 (81)		10 (42)		.004
**Race/ethnicity, n (%)**							
	Black, non-Hispanic	14 (39)		11 (46)		.92	
	White, non-Hispanic	17 (47)		12 (50)			
	Hispanic or Latino	2 (6)		0 (0)			
	Other	3 (8)		1 (4)			
**Education level, n (%)**							
	No college degree	22 (61)		14 (58)		.99	
	College degree	14 (39)		10 (42)			
**Annual income (US $), n (%)**							
	<50,000	13 (36)		9 (38)		.66	
	50,000-100,000	14 (39)		7 (29)			
	>100,000	9 (25)		8 (33)			
Length of stay (days), mean (SD)			4.4 (2.4)		4.1 (2.1)		.56
Charlson Comorbidity Index, median (IQR)			2 [0]		1 [0]		.01
BMI (kg/m^2^), mean (SD)			28.4 (8.1)		27.0 (6.8)		.48
Baseline steps per day			3032 (1201)		5417 (2561)		<.001
**Baseline standardized assessments**							
	Activities of daily living^a^	4.7 (0.8)		4.5 (1.1)		0.61	
	Instrumental activities of daily living^b^	7.6 (1.1)		7.7 (1.1)		0.60	
	Life Space Assessment^c^	80.1 (32.1)		85.7 (27.9)		0.47	
	Short Form-12 physical component score^d^	34.7 (10.5)		36.0 (9.9)		0.62	
	Edmonton Symptom Assessment^e^	30.1 (15.2)		27.2 (13.4)		0.44	
	Pittsburgh Sleep Quality Index^f^	7.3 (3.8)		5.5 (3.0)		0.04	

^a^Scores range from 0 to 6; higher scores indicate greater independence.

^b^Scores range from 0 to 8; higher scores indicate greater independence.

^c^Scores range from 0 to 120; higher scores indicate greater community mobility.

^d^Scores range from 0 to 100; higher scores indicate greater physical health.

^e^Scores range from 0 to 80; higher scores indicate greater symptom burden.

^f^Scores range from 0 to 21; higher scores indicate worse sleep quality.

Less active participants (n=36) had a mean age of 41.7 (SD 13.5) years and a mean hospital length of stay of 4.4 (SD 2.4) days; in addition, 81% (29/36) were female (Table 1). Active participants (n=24) were significantly younger with a mean age of 29.8 (SD 10.9) years and a mean hospital length of stay of 4.1 (SD 2.1) days; 42% (10/24) were female. Both groups reported poor sleep quality at baseline. The less active group had a mean of 3032 (SD 1201) steps at baseline, which was significantly less than the active group, at 5417 (SD 2561) steps.

**Figure 4:**
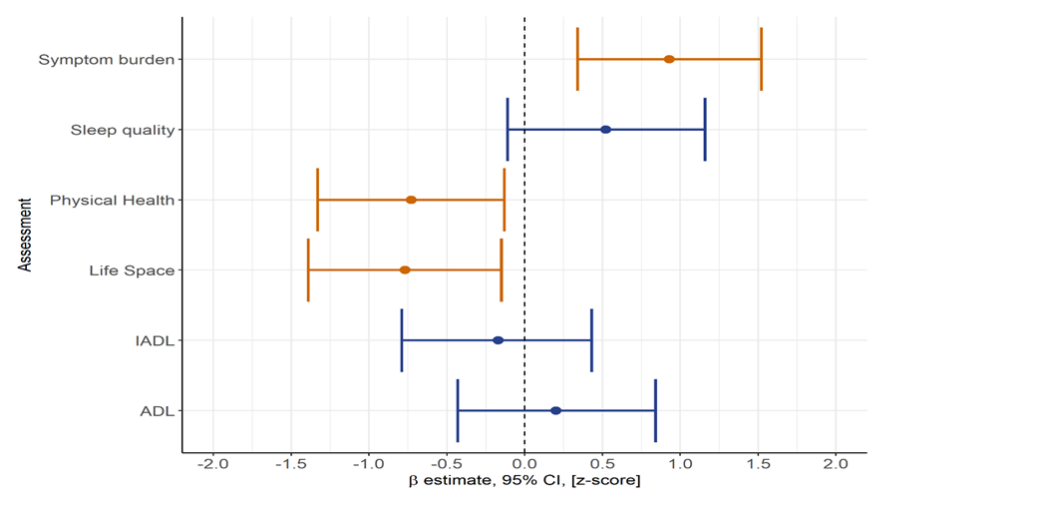
Beta estimates, with 95% CIs, at 13 weeks for the less active cohort; orange bars indicate significant (*P*<.05) comparisons, while the blue bars indicate nonsignificant comparisons. ADL: activities of daily living; IADL: instrumental activities of daily living.

As shown in Figure 4, compared with active participants, less active participants reported significantly less life space mobility (z-score –0.77, 95% CI –1.3 to –0.15; *P*=.02), poorer physical health (z-score –0.73, 95% CI –1.3 to –0.13; *P*<.003), and significantly higher symptom burden (z-score 0.93, 95% CI 0.3 to 1.5; *P*=.02) at 13 weeks postdischarge. These differences were not observed at baseline (Table 1).

## Discussion

### Principal Findings

Consistent with prior studies that have found a parabolic relationship [42] between sleep and worse outcomes such as cardiovascular events [43] or mortality [44], we found physical activity after hospital discharge was worst among those with either too much or too little sleep after hospital discharge. By restricting our sample to exclude those with too much or too little and focusing on those who slept the recommended amount (average 7-9 hours/night) [32,33], we were able to disentangle sleep and physical activity as an initial step to better understand the “sweet spot” of these 2 behaviors on longer-term patient-reported outcomes. In a subgroup of participants who averaged the recommended 7-9 hours of sleep a night, those who were less active (<5000 steps/day) reported significantly reduced life space mobility, physical health, and increased symptom burden, compared with those who were active (>5000 steps/day), at 13 weeks after hospitalization. These significant differences were not observed at baseline but emerged over time, suggesting that prolonged, low levels of physical activity over 13 weeks after discharge from the hospital are associated with worse patient-reported outcomes and may hinder functional recovery. Conventional wisdom might suggest that patients recovering from hospitalization should focus on rest to maximize functional recovery (ie, sleep 8-10 hours rather than 7-9 hours and walk less than 5000 steps), but our results suggest a shift in focus: It may be more important to focus on the right balance of sleep and activity rather than to maximize rest.

Our findings are especially striking considering that our sample was relatively young (mean age 40 years) and healthy (Charlson Comorbidity Index score range: 1-2) compared with a typical population of adults hospitalized for general medicine services. Although this likely represents selection bias (see the Limitations section) for our sample, we were surprised by these findings in a relatively young and healthy cohort. The relationship between sedentary levels of physical activity and adverse outcomes in older, multimorbid adults is very well-known, especially during and after acute illness [45,46]. Indeed, posthospital syndrome was first described in the Medicare population, and low mobility in this population has been described as “toxic” [47] and “epidemic” [48]. It is thus likely that the effects described here may occur at even lower thresholds, below 5000 steps/day, in older adults with greater comorbidity. This represents an important and testable hypothesis for future studies leveraging mHealth technologies. Furthermore, although studies with older adults have not suggested any differential impact of low levels of physical activity by gender, we observed that patients who were less active in the postdischarge period were more likely to be middle-aged women. We did not observe differences by race/ethnicity, income, education, or BMI. Our findings suggest that posthospital syndrome may impact younger and healthier patients who may be considered low risk by clinicians, and middle-aged women in particular may be at higher risk of being overlooked. These are also testable hypotheses for future mHealth studies with larger enrollment.

These findings add to the growing body of literature exploring the effects of sleep and physical activity on functional outcomes after hospital discharge. Previous work has primarily focused on older adults or critically ill patients [4,5,13,49], while this study’s population was younger and admitted to a general medicine or oncology service. Compared with older adults and critically ill patients, those who are younger and without critical illness may have greater ability to return to normal sleep and activity patterns after discharge. However, in our cohort of younger adults without critical illness and adequate sleep, sedentary activity appears to be associated with worse functional outcomes. This provides empirical data to support the application of geriatric paradigms for sleep, activity, and other aspects of transitions of care to a broader (nongeriatric) population [50,51].

### Limitations

Several limitations should be considered when interpreting these data. We examined patient-reported outcomes at a single time point. First, given that our sample was younger and healthier than the general population of hospitalized patients, it is likely that older and sicker patients opted to wear their devices less consistently, thus limiting our ability to describe the effects of poor sleep or low activity levels on this vulnerable population. Discontinuation of device use among high-risk populations after discharge has been observed in other studies and represents an important challenge for the field of mHealth [52]. Second, these cross-sectional findings do not provide insight into when the observed differences in patient-reported outcomes emerged over the 13-week period, and future work may want to investigate this understudied temporal relationship. Third, the small sample size limits generalizability; therefore, our results should be viewed as hypothesis-generating and inform future, larger studies examining these complex constructs. Fourth, we did not have in-hospital sleep and activity data for this sample, limiting our ability to examine the impact of in-hospital sleep and physical activity on longer-term outcomes and the potential contribution to recovery. Last, device-measured prehospital activity levels (steps per day) and sleep levels (hours per night) were unknown, and without these data, we were unable to examine if participants’ posthospitalization sleep and activity patterns differed from prehospitalization patterns.

### Conclusion

In conclusion, these findings are an important step in understanding how posthospitalization sleep and physical activity impact longer-term functional outcomes. Participants within the “sweet spot” that balances recommended sleep (7-9 hours/night) and physical activity (>5000 steps/day) reported better functional outcomes after 13 weeks compared with participants outside the “sweet spot.” Future interventions to improve functional outcomes posthospitalization should leverage wearable devices to further explore the effects of the “sweet spot” of sleep and activity on functional outcomes.

## Abbreviations

CART: classification and regression tree
mHealth: mobile health
MOVE IT: Mobility and Outcomes for Validated Evidence Incentives Trial
PCS-12: Short-Form 12 physical component scale
PSQI: Pittsburgh Sleep Quality Index
